# Characteristics of plasma sheath in multi-component plasmas with three-ion species

**DOI:** 10.1038/s41598-022-10838-0

**Published:** 2022-04-28

**Authors:** M. M. Hatami, I. Kourakis

**Affiliations:** 1 Physics Department, K. N. Toosi University of Technology, Tehran, 15418-49611 Iran; 2 Mathematics Department, College of Science and Engineering, Khalifa University of Science and Technology, P.O. Box 127788, Abu Dhabi, UAE

**Keywords:** Plasma physics, Astrophysical plasmas

## Abstract

The plasma sheath of a three ion species plasma is studied numerically, relying on the results of the experiment by Yip et al. (Phys. Plasmas 23:050703 (2016) to measure the positive ion velocities at the sheath edge. The positive ion species ($Ar^+$, $Kr^+,$ and $Xe^+$) are assumed to be singly charged and to be characterized by the same temperature. It is shown that the sheath characteristics, viz. the particle number densities, the electrostatic potential and the space charge density profile in the sheath all depend on the $Kr^+$ concentration that is gradually added to the argon-xenon plasma as the third positive ion species. Also, the effect of ion-neutral collisions on the sheath properties is investigated numerically. Our results may be extended to a multi-ion plasma with more than two species of positive ions.

## Introduction

Plasmas are large ensembles of charged particles (ionized gases), overall quasi-neutral in principle, which either occur in various forms in nature or may be produced artificially in the laboratory. In the laboratory, plasmas are fabricated in large chambers hosting electric discharge experiments, in which the plasma is separated from the wall surface by a thin positively charged region called *the sheath*. This arises from the difference in mobility between the ions and the electrons. Although plasma sheath formation is one of the oldest problems in plasma physics, it is still far from being thoroughly understood and attracts attention among researchers, due to its importance in the modification of the surface properties of the materials, in excitation of electrostatic waves and also due to its relevance in magnetic confinement fusion plasma^1,2^.

Bohm was the first to show that there exists a necessary condition for the formation of the plasma sheath in a cold electron-ion plasma^3^. The so called Bohm criterion imposes $v_{0, i}\ge c_s$ where $v_{0, i}$ is the drift velocity of the positive ions directed towards the wall at the sheath edge, $c_s=(k_BT_e/m_i)^{1/2}$ is the ion acoustic velocity (the sound speed), $T_e$ is the electron temperature, $k_B$ is the Boltzmann constant and $m_i$ is the ion mass. Following this paradigm, many attempts have been made to modify the Bohm criterion to include other plasma parameters such as the ion temperature, the ion-neutral collision frequency, the ambient magnetic field etc.; see for instance in Refs.^4–6^.

Many authors have studied the sheath region of multi-component plasmas, i.e. plasmas containing electrons and several species of positive and (possibly) negative ions, under different conditions by using fluid modeling. A plasma sheath in an electropositive plasma with two types of positive ions has been investigated by Franklin^7^. Furthermore, Shaw *et al* studied sheath dynamics in a magnetized electronegative plasma consisting of two species of warm positive ions, negative ions and electrons^8^. The effect of ion-neutral collisions on the sheath dynamics in unmagnetized electronegative plasma with two types of positive ions was investigated by Moulick et al^9,10^. Moreover, Fouial *et al* explored the effect of non-thermal electrons and dust grains on the sheath region of an unmagnetized plasma containing of argon and helium positive ions^11^.

Multi-component plasmas are observed in many real plasmas, such as electron emission gun plasmas or in vacuum arc plasma deposition systems. Multi-species plasmas may also be produced by plasma-wall interaction. The study of the structure and characteristics of boundary layers of such multiple-ion species plasmas is of great importance in understanding the effect of charged particles on substrates and solid walls. The first step in this study is to determine the Bohm criterion in multi-species plasmas. Because of the importance of this topic, many authors have attempted to determine the sheath formation criterion of multi-component plasmas and then to investigate the sheath properties in such plasmas^12–18^. Among these fundamental studies, we distinguish Riemann’s^12^, who derived a generalized Bohm criterion for a multi-component plasma as follows:

1 $$\begin{aligned} \sum _i \displaystyle \frac{n_{0i}c_{si}^2}{n_{0e}v_{0i}^2}\le 1, \end{aligned}$$

where $v_{0i}$ and $c_{si}=(k_BT_e/m_i)^{1/2}$ are the individual drift and sound velocity of each of the ion species, while $n_{0i}$ and $n_{0e}$ are the ion and electron densities at the sheath boundary, respectively. From (1), it is clear that unlike the case of a single species plasma where $v_{0i}\ge c_s$, the equality may be satisfied by speeds faster or slower than the individual sound speed corresponding to a (any) given ion species among the various ion species present in the plasma.

Unlike an electron-ion plasma (i.e. with a single ion species), inequality (1) does not uniquely determine the flow speed of each ion species at the sheath edge if there is more than one species of ion. For instance, in a two ion species plasma, when the electron temperature far exceeds the ion temperature ($T_e \gg T_i$), two possibilities exist for (1) to be satisfied^18^: either (i) all ions reach the sheath edge with a common velocity, called “the system sound speed” $c_{sys}=\left( \sum _i n_{0i}k_BT_e/n_{0e}m_i\right) ^{1/2}$, or (ii) each of the ion species has its own Bohm velocity at the sheath edge. Unlike the theoretical prediction^14^, experimental results for two-ion species plasmas did not confirm the latter solution but instead showed that the ion speed at the sheath boundary is closer to the system velocity, $c_{sys},$^4,15–24^. In most of these experiments, the laser-induced fluorescence (LIF) technique was used to measure the velocity of each ion species at the sheath edge. More experimental evidence for the system sound speed solution has been provided by Lee *et al.*^15^. They studied the Bohm criterion in single and two ion species plasmas with LIF in *Xe* and $Ar-Xe$ plasmas and showed that the argon and xenon ion velocities approach the ion sound speed of the system near the sheath boundary. Oksuz *et al.*^16^ used electrostatic probes and ion-acoustic waves to measure the drift speed of ions in the plasma-boundary transition region. They measured the ion-acoustic wave speed at the sheath edge to be twice what it is in the bulk plasma in a two-ion-species plasma. None of these experiments provided a physical mechanism for these observations. On the other hand, theoretical and experimental investigations have shown that if the plasma contains multiple ion species, ion-ion two-stream instabilities can be expected to arise independently, in addition to ion-acoustic instabilities in the presheath under conditions of low neutral pressure and $T_e/T_i \gg 1$ due to convection of one species onto another^17,19,23–25^. Following these considerations, it appears that these flow-driven instabilities may play an important role in the determination of the ionic speeds at the edge of the plasma sheath. In 2009, Baalrud *et al*^17^ showed that considering collisional friction enhanced by ion-ion streaming instabilities explained the discrepancy in the latter experiment. They showed that the collisional friction force causes the faster ion species to slow down and the slower species to accelerate. Considering this fact, they determined the solution of the plasma sheath criterion in two ion species plasmas. In fact, the theory suggested by Baalrud et al^17^ indicated that the difference in velocities at the sheath edge depends on the relative concentrations of the two ions as follows: the difference in velocities is small, with both species approaching the bulk sound velocity, when the concentrations are comparable, and becomes large, with each species reaching its own Bohm velocity, when the relative concentration difference becomes significant. To test these findings, drift velocities of *Ar* and *Xe* ions were measured with the LIF technique^18^. The predictions were found to be in excellent agreement with the experimental data.

Although it might appear that, in the light of these experimental observations, the dynamics related to the Bohm criterion in multi-ion plasmas has been elucidated, a recent experiment by Yip et al.^26^ showed that despite being one of the oldest problems in plasma physics, sheath formation and its criterion are still not fully understood. For the first time, they measured the drift velocities of two ion species at the sheath boundary of a three-ion component plasmas and found an unexpected feature regarding the ion speeds at the sheath edge, when there are three positive ion species: under most circumstances, the speed at which the ions enter into the sheath region matches neither the “system” sound speed nor their individual Bohm speed. Their experimental results demonstrated that if an additional third ion component was added to a two-ion plasma, the drift velocity (value) of each of the main two ion species at the sheath edge iterates from the system sound speed to their individual Bohm velocity as the concentration of the additional (third) ion species increases.

The aforementioned surprising findings of Yip et al.^26^ work have motivated us to numerically investigate the sheath characteristics in an electropositive plasma with three ion species. We have undertaken a meticulous study of the spatial profile(s) of the (various) ion density, electrostatic potential and space charge density in a multi-component plasma consisting of singly charged $Ar^+$, $Kr^+$ and $Xe^+$ ions. Our results show that the presence of the third positive ion species increases the sheath potential but decreases the space charge and the sheath thickness. We combine recent results based on kinetic modeling with experimental results on three-component plasma to accurately compute the velocity of the ion species at the sheath edge which are the necessary initial conditions in our model. This allows us to investigate the effect of ion-neutral drag forces on the structure and to characterize the properties of the plasma sheath in a plasma containing three positive ions species, an opportunity offered for the first time, to the best of our knowledge.

This study is organized in four sections. Following this Introduction (Sect. 1), the basic equations of our model are laid out in Sect. 2. In Sect. 3, the model equations are solved numerically and the obtained results are analyzed. Our conclusions are eventually summarized in the final Sect. 4.

## Model and basic equations

An unmagnetized electropositive plasma consisting of electrons and three types of singly charged positive ions with different masses and the same temperatures is considered. The electrons are assumed to be in thermal equilibrium, hence the electron density obeys the Boltzmann distribution^4,5,14^. Furthermore, the plasma is assumed to be in contact with a planar surface perpendicular to the *x*-axis and to have reached the steady state. Moreover, it is assumed that the physical parameters of the sheath vary only along the perpendicular direction to the surface (*x*-direction).

Under the steady state condition and ignoring further ionization resulting in extra source/sink terms, the (stationary form of the) governing equations for each of the ion species will include the fluid continuity and momentum equations:

2 $$\begin{aligned} \frac{d}{dx}(n_{i}v_{i})= & {} 0, \end{aligned}$$

3 $$\begin{aligned} m_{i}v_{i}\frac{d v_{i}}{dx}= & {} -e\frac{d\varphi }{dx}-\frac{1}{n_i}\frac{d p_i}{dx}-m_i\nu _{i}v_i, \end{aligned}$$

where *x* is the distance from the sheath edge, $n_{i}$, $v_{i}$, $m_{i}$, $p_i=n_ik_BT_{i}$ and $\nu _{i}=n_n\sigma _{s,i} v_i$ are the density, velocity, mass, pressure and the effective ion-neutral collision frequency of the *i*th ion species ($i=1,2,3$), respectively, $n_n$ is the neutral gas density and $\sigma _{s,i}$ is the momentum transfer cross section for collisions between each ion species and neutrals. The coupled fluid equations are closed by Poisson’s equation which reads:

4 $$\begin{aligned} \frac{d^2\varphi }{dx^2}=-\frac{e}{\varepsilon _{0}}\bigg (\sum _{i =1} ^3 n_{i}-n_{e}\bigg ). \end{aligned}$$

The quasi-neutrality condition imposed by the equilibrium requirement reads:

5 $$\begin{aligned} \sum _{i =1} ^3 n_{0i}=n_{0e}, \end{aligned}$$

where $n_{0i}$ is the *i*th ion species density at the sheath edge ($x=0$).

It is convenient to introduce dimensionless variables

$$\begin{aligned} \phi= & {} \displaystyle \frac{-e\varphi }{k_BT_e},~~ N_e=\displaystyle \frac{n_e}{n_{0e}},~~ X=\displaystyle \frac{x}{\uplambda _{De}}, \\ u_i= & {} \displaystyle \frac{v_i}{c_{s1}},~~\tau _i=\displaystyle \frac{T_{i}}{T_e},~~N_i=\displaystyle \frac{n_i}{n_{0e}} \end{aligned}$$

where $c_{s1}$ is the sound velocity of the lightest ion species in this work and $\uplambda _{De}=(k_BT_{e}/4\pi e^2n_{0e})^{1/2}$. Using these variables, Eqs. (2)–(5) can be rewritten in dimensionless form as follows:

6 $$\begin{aligned} \left( 1-\frac{\tau _1}{u_1^2}\right) u_{1}\frac{du_{1}}{dX}= & {} \frac{d\phi }{dX}-\alpha _1 u{_1}^2, \end{aligned}$$

7 $$\begin{aligned} \left( 1-\mu _1\frac{\tau _2}{u_2^2}\right) u_{2}\frac{du_{2}}{dX}= & {} \mu _1\frac{d\phi }{dX}-\alpha _2 u{_2}^2, \end{aligned}$$

8 $$\begin{aligned} \left( 1-\mu _2\frac{\tau _3}{u_3^2}\right) u_{3}\frac{du_{3}}{dX}= & {} \mu _2\frac{d\phi }{dX}-\alpha _3 u{_3}^2, \end{aligned}$$

9 $$\begin{aligned} \frac{d^2\phi }{d X^2}= & {} \frac{N_{01}u_{01}}{u_1}+\frac{N_{02}u_{02}}{u_2}+\frac{N_{03}u_{03}}{u_3}-\exp (-\phi ), \end{aligned}$$

where $N_{01}=n_{01}/n_{0e}$, $N_{02}=n_{02}/n_{0e}$, $N_{03}=n_{03}/n_{0e}$, $u_{01}=v_{01}/c_{s1}$, $u_{02}=v_{02}/c_{s1}$, $u_{03}=v_{03}/c_{s1}$, $\mu _1=m_1/m_2$, $\mu _2=m_1/m_3$ and $\alpha _i = n_n \sigma _{s,i} \uplambda _{De}$ ($i = 1, 2, 3$) is a dimensionless collisionality parameter (that differs among the ion species).

For our numerical analysis, we have adopted a standard 4th-order Runge-Kutta method with step-size $h=0.003$ is used to solve Eqs. (6)–(9) with the boundary conditions $u_i(X=0)=u_{0i}$, $N_i(X=0)=N_{0i}$, $\phi (X=0)=0$ and $d\phi /dX=0$ at the sheath edge and $\phi (X)=\phi _w$ on the wall. The differential equations are solved from the sheath edge $(X=0)$ towards the wall. To determine the wall position and hence the sheath thickness, we start from the sheath edge and move in space until the requirement $\phi (X)=\phi _w$ is fulfilled. It should be pointed out here that, following^26^, we have adopted the velocity values reported for the ions at the sheath edge. Therefore, flow-driven instabilities such as ion-ion two-stream instabilities were ignored in our computational model.

## Results and discussion

We will investigate the sheath structure in an electropositive plasma consisting of electrons and singly charged $Ar^+$, $Kr^+$ and $Xe^+$ ions with $T_{e}=1.95~eV$, $T_{1}=T_{2}=T_3=0.03~eV$, $\varphi _w=-90~eV$ and equal concentrations of argon and xenon ions $(n_{0Ar^+}=n_{0Xe^+})$, i.e. reproducing the experimental conditions of ^26^. We assume that krypton is gradually added to the low pressure unmagnetized discharge into a mixture of argon and xenon. In the following, argon, krypton, and xenon ions are labeled as species 1, 2, and 3, respectively. Also, as mentioned in Sec. 3, there are three unspecified parameters $\alpha _1$, $\alpha _2$ and $\alpha _3$ in Eqs. (6)–(8) which depend on the collision cross section of the respective ion species. In the energy range of interest ($\sim eV$), the ion-neutral cross section can be considered to be constant and is taken to be $\sim 5\times 10^{-15} \mathrm{cm}^2$ for argon^1^. We have taken $\alpha _2/\alpha _1\approx 2.5$ and $\alpha _3/\alpha _1\approx 4.5$ in our numerical solution of Eqs. (6)-(8)^1^. Moreover, according to both the experimental data and the theoretical predictions in^26^, the argon and xenon drift velocities at the sheath edge ($v_{01}, v_{03}$) change from a value close to the system sound speed $c_{sys}$ towards their individual sound speeds $c_{si}$ as the krypton concentration increases. Therefore, depending on the value of krypton concentration $N_{02}$, we shall use different values for $v_{01}$ and $v_{03}$ in our calculation. In addition, following the Yip *et al.*^26^ work, we assume that krypton has its individual sound speed at the sheath edge ($v_{02}=c_{s2}$), regardless of its concentration. As mentioned in^26^, this is a simple approximation based on the argument that the instability enhanced friction affects only the argon and xenon ions as the main cause of two-stream instability, i.e. not the krypton ions, which play no role in the occurrence of this instability. Arguably, this theoretical prediction should be tested experimentally.

Considering the above mentioned conditions and parameters, we examine the sheath structure in a multi-component electropositive plasma containing three species of positive ions ($Ar^+$, $Kr^+$ and $Xe^+$) and electrons by studying the dynamical profile of the charged particle density, electric potential and space charge.

**Figure 1:**
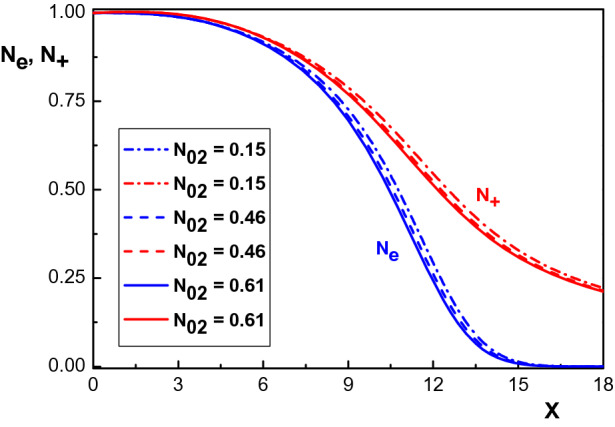
Variation of the normalized density distribution of the net positively charged particles ($N_+$) and electrons ($N_e$) versus the normalized distance from the sheath edge for $\alpha _1=0.001$, $\varphi _w=-90~eV$, for different values of krypton concentration $N_{02}$.

Figure 1 shows the effect of the krypton concentration $N_{02}$ on the density distribution of net positive ($N_+=N_1+N_2+N_3$) and negative ($N_e$) charged particles for $\alpha _1=0.001$, $\varphi _w=-90~eV$ and different values of $N_{02}$. Depending on the krypton concentration, we have used the following values for the Bohm velocity of each ion species in this figure^26^: $v_{01}=1750~m/s$, $v_{02}=1500~ m/s$, $v_{03}=1470~ m/s$ for $N_{02}=0.15$, $v_{01}=1890~m/s$, $v_{02}=1500~ m/s$, $v_{03}=1320~ m/s$ for $N_{02}=0.46$ and $v_{01}=1980~ m/s$, $v_{02}=1500 ~m/s$, $v_{03}=1270 ~m/s$ for $N_{02}=0.61$. Also, following^26^, we assume that the argon and xenon ions have the same concentration $N_{01}=N_{03}$. From Fig. 1, it is seen that the density distribution of the electrons as well as that of the net positive ions $N_+$ both decrease in space upon increasing the krypton concentration.

**Figure 2:**
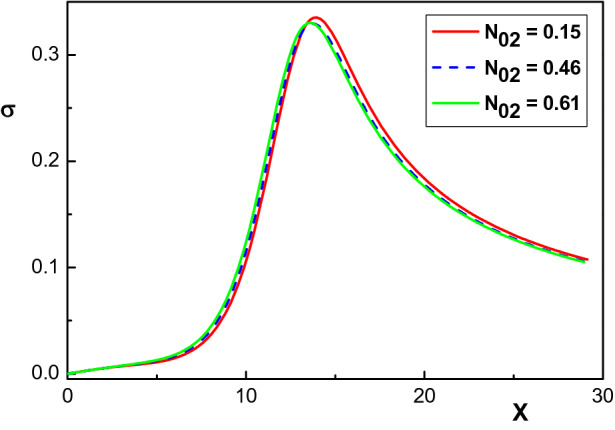
Variation of the normalized space charge $\sigma$ versus the normalized distance from the sheath edge for different krypton concentrations. The other parameters are the same with Fig. 1.

The evolution of the space charge $\sigma =N_+-N_e$ inside the sheath region is depicted in Fig. 2 for three different values of the krypton concentration: $N_{02}=0.15$, $N_{02}=0.46$ and $N_{02}=0.61$. The other parameters are the same as in Fig. 1. Similar to Fig. 1, it is seen that an increase of the initial density of krypton ions causes the space charge in the sheath region to decrease. It is also observed that, with increasing $N_{02}$, the density of electrons drops to zero more rapidly, whereas the positive ion densities drop slowly at the beginning. Therefore, the space charge emerges at a peak value which indicates that in this region, more positive particles gather to shield the negative potential of the board. Figure 2 shows that this peak shifts towards the sheath edge with increasing $N_{02}$. Moreover, it can be seen from this figure that the sheath thickness (values), the distance between the sheath edge $(X=0)$ and also the point where the condition $\phi =\phi _w$ is fulfilled, are all sensitive to the presence of krypton ions and in fact decrease as $N_{02}$ increases.

**Figure 3:**
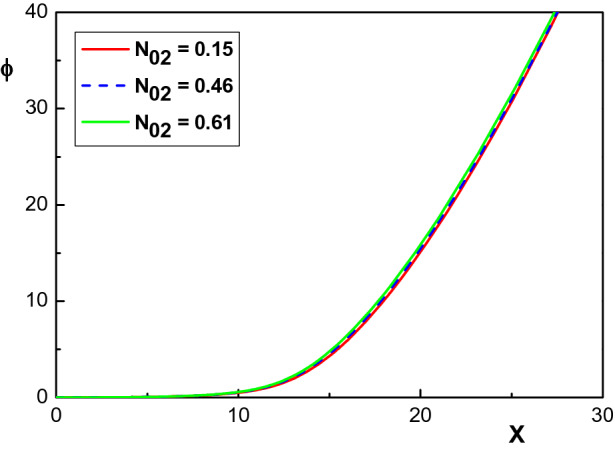
Variation of the normalized electrostatic potential $\phi$ versus the normalized distance from the sheath edge for different concentrations of krypton ions and the same parameters of Fig. 1.

Figure 3 shows the variation of the normalized electrostatic potential in the sheath region of $Ar^+-Kr^+-Xe^+$ plasma for two different concentrations of krypton ions and for the same parameters as in Fig. 1. It is observed that the normalized potential of the sheath decreases infinitesimally by increasing $N_{02}$. This is consistent with the result of Fig. 2, which showed that the space charge decreases with increasing krypton concentration. The independence of the sheath width from the ion mass is another point seen in this figure, which is in agreement with the results of Fig. 2 as well as Child-Langmuir law^1^.

**Figure 4:**
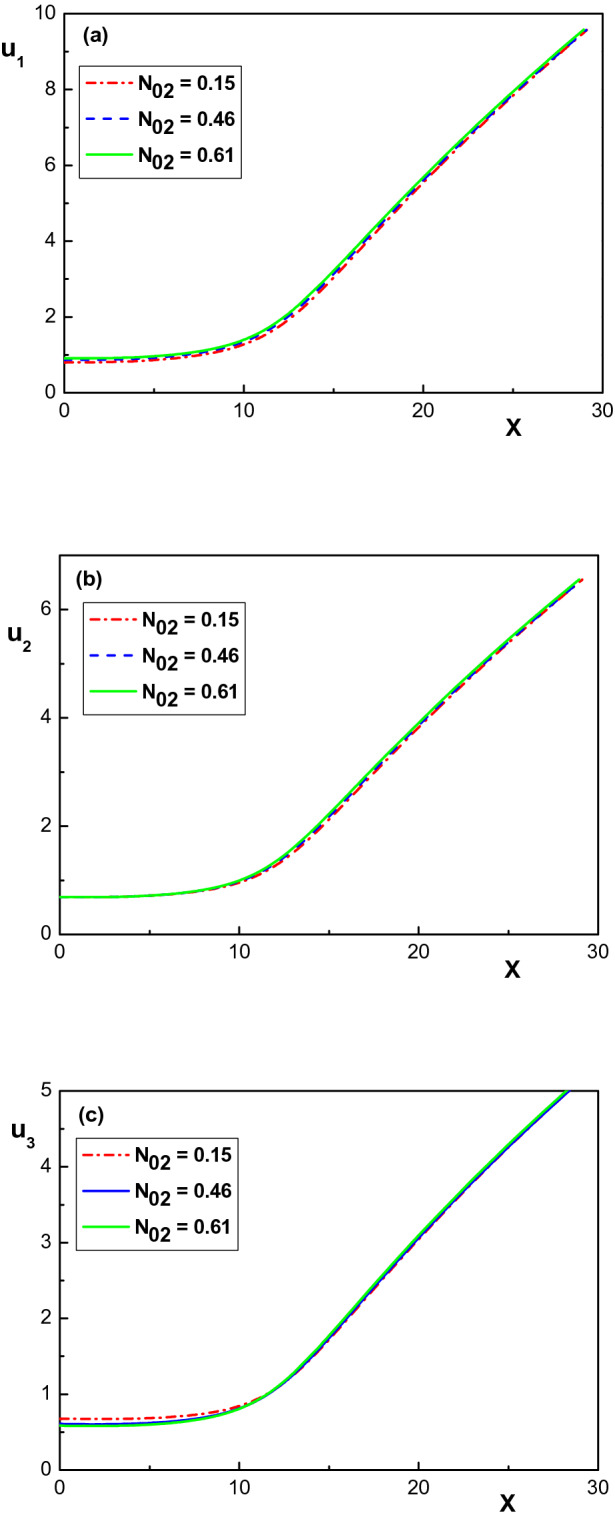
Variation of the normalized ion velocities versus the normalized distance from the sheath edge for different values of $N_{02}$. The other parameters are the same with Fig. 1.

Figure 4 shows the variation of the ion velocities throughout the sheath layer for different values of $N_{02}$. The other parameters are the same as in Fig. 1. It is seen that an increase in $Kr^+$ concentration leads to an increase in the velocity of all ion species in the sheath region. Therefore, it is obvious from Figs. 1, 2, 3 and 4 that the krypton ion presence affects the profiles of the electrostatic potential, velocity and charged particle density distribution and the space charge of a three ion species plasma in a significant way.

In the light of the two main assumptions considered by Yip et al^26^, namely that the krypton velocity and the density of argon and xenon remain constant despite the increase in krypton concentration, which we have also adopted in our present work, but also in the absence of further experimental results, it would take more time to provide conclusive explanations for the changes observed as the krypton concentration increases; this task is therefore outside our scope for now.

**Figure 5:**
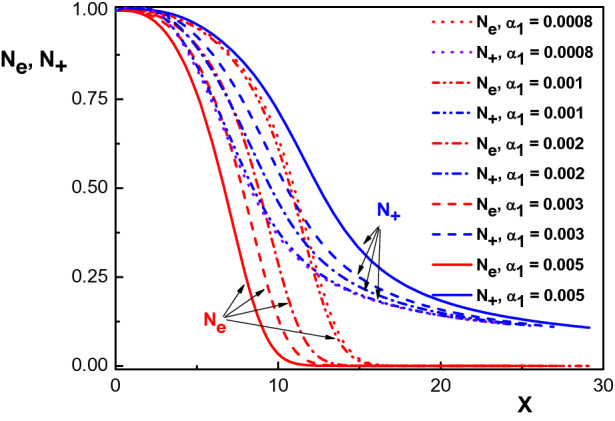
Variation of the normalized density distribution of the net positive charged particles ($N_+$) and electrons ($N_e$) versus the normalized distance from the sheath edge for $N_{02}=0.15$, $v_{01}=1750~m/s$, $v_{02}=1500~m/s$, $v_{03}=1470~ m/s$ and different values of $\alpha _1$. The other parameters are the same with Fig. 1.

We shall now investigate the effect of the ion-neutral drag force on the sheath dynamics. To do this, we will solve the basic equations of our model for different $\alpha _1$ values. The effect of the drag force on the density distribution of the electrons and of the net positive ion species is shown in Fig. 5 for $N_{02}=0.15$, $v_{01}=1750~m/s$, $v_{02}=1500~ m/s$, $v_{03}=1470~ m/s$, for different values of $\alpha _1$. This graph indicates that unlike the ion density distribution, the falloff of the density distribution of electrons through the sheath becomes faster as the drag force increases. In addition, similar to Refs.^27–29^, the decrease in sheath thickness due to the increase in $\alpha _1$ is clearly visible in this figure. Moreover, it is seen from Fig. 5 that for $\alpha _1 < 0.001$ the results are reduced to the collisionless case, within good approximation.

**Figure 6:**
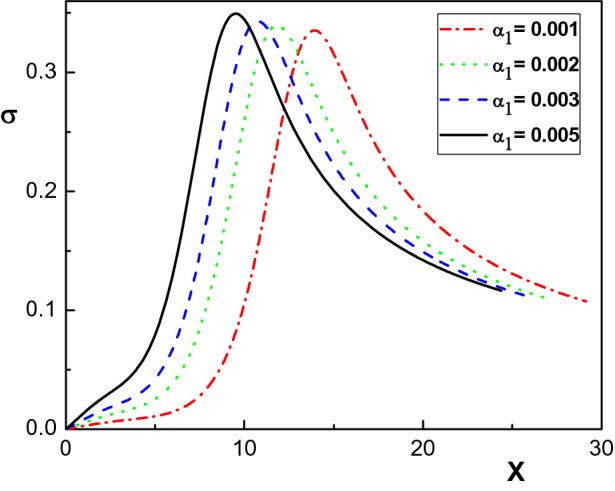
Variation of the normalized space charge $\sigma$ versus the normalized distance from the sheath edge, for different values of $\alpha _1$ and for the same parameters as in Fig. 4.

In Fig. 6 the dependence of the space charge density distribution on the ion-neutral drag force is demonstrated. From this plot, we see that an increase in the ion-neutral collision frequency leads to an increase in the space charge peak due to the reduction in positive ion velocity. In addition, by increasing the collisional effects (via increasing $\alpha _1$), the charge accumulation position shifts towards the sheath edge.

**Figure 7:**
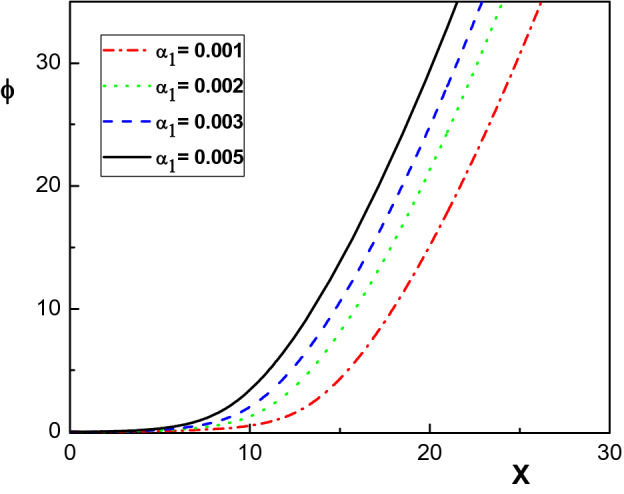
Variation of the normalized electrostatic potential $\phi$ versus the normalized distance from the sheath edge, for different values of $\alpha _1$ and for the same parameters as in Fig. 4.

**Figure 8:**
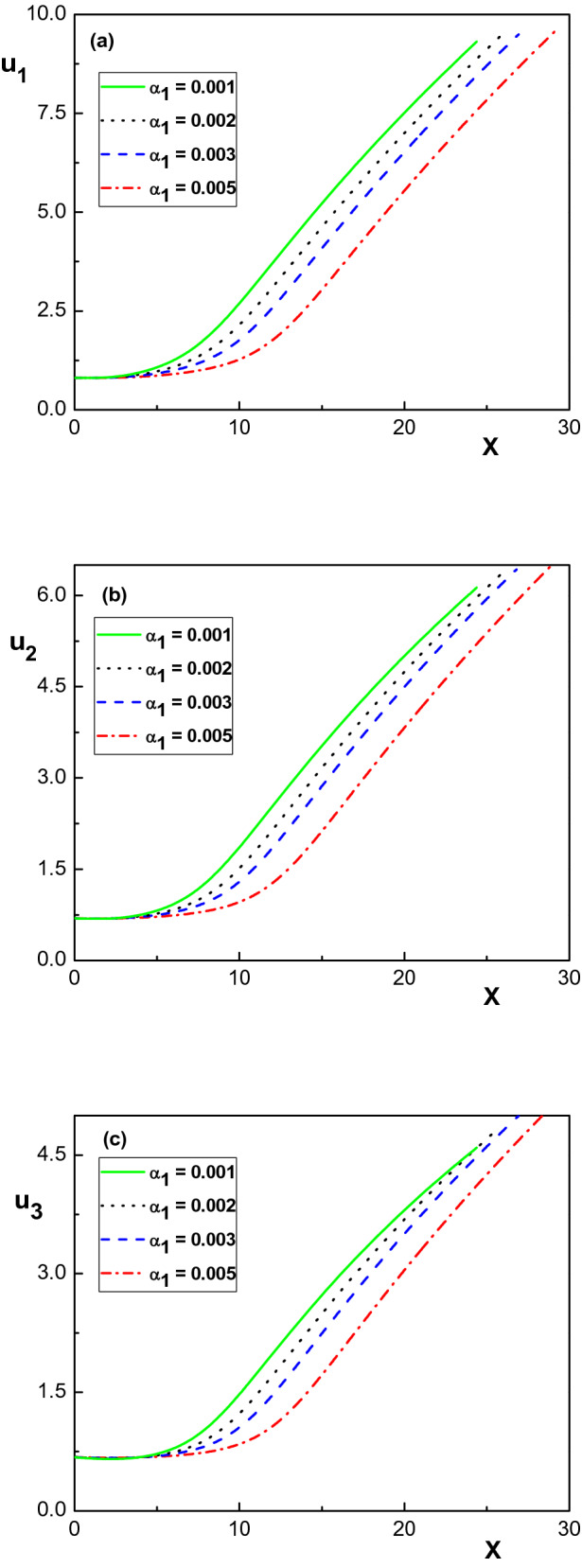
Variation of the normalized ion velocities versus the normalized distance from the sheath edge for different values of $\alpha _1$. The same parameters as in Fig. 4.

Figure 7 illustrates the effect of the collision force on the electrostatic potential. Similar to plasmas with two positive ion species^29^, it is seen that the electrostatic potential (the sheath thickness) increases (decreases) by increasing the ion-neutral drag force (increasing $\alpha _1$). Taking into account the variation of the space charge with increasing $\alpha _1$, the behavior of $\phi$ in Fig. 7 is reasonable. We conclude that the dynamical properties of the sheath depend considerably on the ion-neutral drag force.

Finally, Fig. 8 shows the variation of the velocity of the ion species throughout the sheath layer with the drag force. From this figure, it is observed that the ion velocities decrease by increasing $\alpha _1$, which is in agreement with the results of previous works for plasmas containing either one or two ion species^10,11,27,29^. Moreover, it is seen that the lighter ion population has higher velocity towards the sheath wall than the heavier ion species.

## Conclusion

Relying on the results of a recent experiment in attempt to evaluate the Bohm velocity of positive ions at the sheath edge of $Ar^+-Kr^+-Xe^+$ plasma, we have investigated the behavior of the electrostatic potential of the density distribution of the charged particles in the sheath region of an electropositive plasma consisting of electrons and three ion species consisting of argon, krypton and xenon positive ions. All types of ions were assumed to be singly charged and to be characterized by a finite temperature. Following the architecture of the experiment, we have gradually increased the krypton concentration in the argon-xenon plasma and we have investigated the impact on the properties and on the structure of the sheath. Our results have shown that the presence of the krypton positive ion species affects the sheath structure and the density distribution profiles of the charged particles. It was found that an increase in $Kr^+$ concentration causes a decrease in the space charge density, which leads to a decrease in the sheath thickness and an increase in the sheath potential. It was also shown that the amplitude of the space charge density peak decreases and shifts towards the sheath edge as $Kr^+$ concentration increases. Moreover, we have shown that the ion-neutral drag force considerably affects the sheath potential and the density distribution of the charged particles. The space charge peak grows with increasing the collision force, and hence the sheath width decreases. In addition, collisional effects cause the amplitude of the space charge peak to increase and its position comes closer to the sheath edge. It was also shown that the velocity of the positive ion species in the sheath layer decreases by increasing the collision force.
